# Norm-Based Coding of Voice Identity in Human Auditory Cortex

**DOI:** 10.1016/j.cub.2013.04.055

**Published:** 2013-06-17

**Authors:** Marianne Latinus, Phil McAleer, Patricia E.G. Bestelmeyer, Pascal Belin

**Affiliations:** 1 Institute of Neuroscience and Psychology, University of Glasgow, Glasgow G12 8QB, Scotland; 2 Department of Psychological and Brain Sciences, Indiana University, Bloomington, IN 47405, USA; 3 School of Psychology, Bangor University, Bangor, Gwynedd LL57 2AS, UK; 4 Département de Psychologie, Université de Montréal, Montréal, QC H2V 2S9, Canada; 5 Institut des Neurosciences de La Timone, UMR 7289, CNRS & Université Aix-Marseille, 13005 Marseille, France

## Abstract

Listeners exploit small interindividual variations around a generic acoustical structure to discriminate and identify individuals from their voice—a key requirement for social interactions. The human brain contains temporal voice areas (TVA) [1] involved in an acoustic-based representation of voice identity [2, 3, 4, 5, 6], but the underlying coding mechanisms remain unknown. Indirect evidence suggests that identity representation in these areas could rely on a norm-based coding mechanism [4, 7, 8, 9, 10, 11]. Here, we show by using fMRI that voice identity is coded in the TVA as a function of acoustical distance to two internal voice prototypes (one male, one female)—approximated here by averaging a large number of same-gender voices by using morphing [12]. Voices more distant from their prototype are perceived as more distinctive and elicit greater neuronal activity in voice-sensitive cortex than closer voices—a phenomenon not merely explained by neuronal adaptation [13, 14]. Moreover, explicit manipulations of distance-to-mean by morphing voices toward (or away from) their prototype elicit reduced (or enhanced) neuronal activity. These results indicate that voice-sensitive cortex integrates relevant acoustical features into a complex representation referenced to idealized male and female voice prototypes. More generally, they shed light on remarkable similarities in cerebral representations of facial and vocal identity.

## Highlights

• Identity coding in temporal voice area in terms of acoustical distance to prototypes
• Description of the “voice space” in terms of simple acoustical measures
• Male and female prototypes are ideally smooth versions of the population means
• Comparable coding mechanism for identity across sensory modalities

## Results

**Figure 1:**
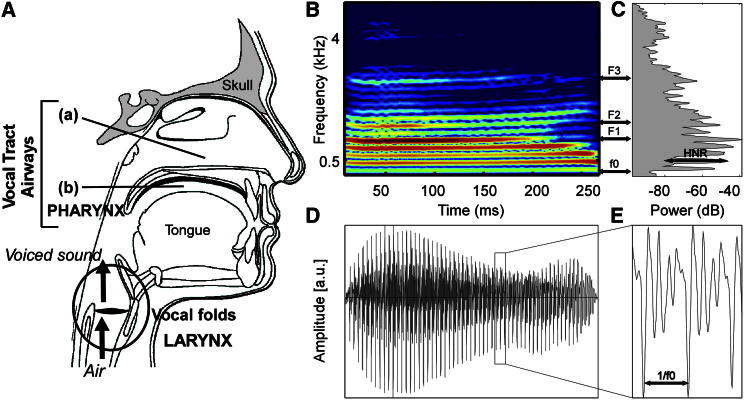
Acoustical Dimensions of Voices (A) During voice production, the vocal folds in the larynx oscillate periodically generating a buzzing sound with a fundamental frequency (f0) and a highly harmonic structure. Acoustical filtering by the vocal tract airways—nasal cavity (a) and mouth cavity (b)—above the larynx modifies this buzzing sound, resulting in regions of enhanced energy in the spectrum called formants. (B) Spectrogram of the syllable “had” spoken by an adult female speaker. Color scale indicates power (dB). Note the vertical stripes corresponding to the harmonics (integer multiples of f0) and the bands corresponding to the formants (F1–F3). (C) Stimulus power spectrum. (D and E) Stimulus amplitude waveform. See also Figure S1 and Table S1 for more information on the acoustical parameters measured in the different studies.

**Figure 2:**
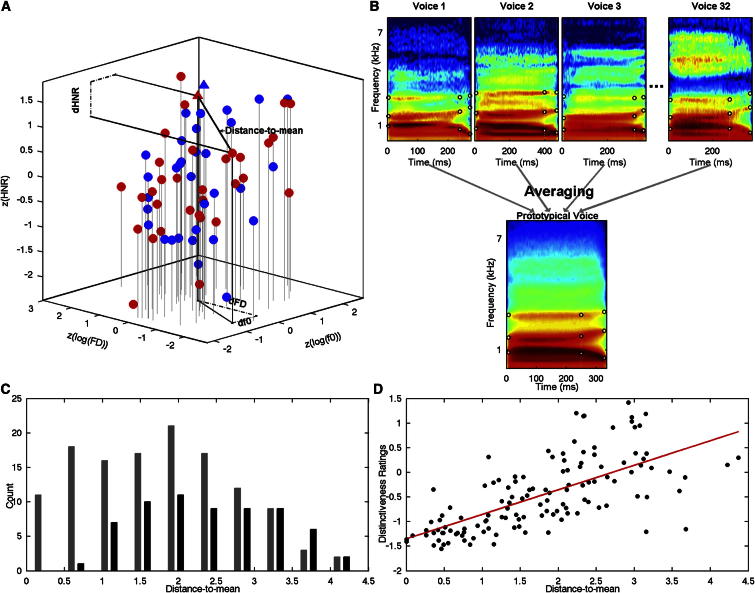
Distance-to-Mean in Voice Space (A) Stimuli from Experiment 1 (32 natural voices per gender) are represented as individual points in the three-dimensional space defined by their average log(f0), log(FD), and HNR, *Z* scored by gender (resulting in overlapping male and female stimulus clouds). Red discs represent female voices; blue discs represent male voices. The prototypical voices generated by averaging together all same-gender stimuli are located on top of the stimulus cloud (triangles) owing to their high HNR value. Distance-to-mean *=*$\sqrt{df0^{2}+dHNR^{2}+dFD^{2}}$. (B) Voice averaging in Experiment 1. Spectrograms of example voice stimuli (top row) represent male speakers uttering the syllable “had.” Black circles indicate manually identified time-frequency landmarks put in correspondence across stimuli during averaging, corresponding to the frequencies of the first three formants at onset of phonation (left side), at onset of formant transition, and at offset of phonation (right side). A prototypical voice (bottom) is generated by morphing together stimuli from 32 different speakers. Note the smooth texture caused by averaging, resulting in high HNR values. (C) Histograms of distance-to-mean distributions for the voice stimuli of Experiment 1 (gray) and Experiment 2 (black); the mode of the two distributions is for intermediate values of distance-to-mean. (D) Scatterplot of distance-to-mean versus distinctiveness ratings (*Z* scored) for the 126 stimuli of Experiment 1. Distance-to-mean explains over half of the variance in distinctiveness ratings (R^2^ = 0.53): voices with greater distance-to-mean are judged to be more distinctive. See also Figure S2 for correlations coefficients in other spaces.

Two difficulties arise when approaching the complex problem of voice identity representation: the high dimensionality of the “voice space,” i.e., the large number of acoustical dimensions potentially differentiating speakers, and the dynamic, ever-changing nature of voices. We examined a simpler version of the problem by first using brief syllable stimuli for which the influence of time is minimal and by focusing on a small number of key acoustical measures. Three acoustical dimensions were selected based on their relevance to voice production and perception [11, 15, 16, 17, 18, 19, 20, 21, 22, 23] (Figure 1; see also Figure S1 available online): the fundamental frequency of phonation (f0, related to the pitch of voice), formant dispersion (FD, the average frequency difference between formants, related to vocal tract size [17]), and the harmonics-to-noise ratio (HNR, a measure of spectrotemporal regularity); together they defined a three-dimensional acoustical voice space (Figure 2A).

### Experiment 1

In Experiment 1, recordings from 32 male and 32 female adult speakers uttering the syllable “had” [24] were subjected to a series of acoustical measures (Table S1; Figure S1; Supplemental Experimental Procedures). Each voice stimulus was represented as a point in the voice space with coordinates corresponding to the stimulus’ average f0, FD, and HNR, *Z* scored by gender (Figure 2A). We generated male and female prototypical voice stimuli by averaging all 32 same-gender voices by using morphing (Figure 2B). The resulting prototypical voices were characterized by (gender-specific) average f0 and FD values but high HNR values as averaging smoothed out spectrotemporal irregularities (Figure 2A; Table S1); as shown in Figure 2A, they are not located at the barycenter but rather on top of the voice stimulus clouds. Voice composites were also generated for each gender by averaging 2 (n = 16), 4 (n = 8), 8 (n = 4), and 16 (n = 2) different voice stimuli, for a total of 126 stimuli. The Euclidean distance between each stimulus and the gender-specific prototypical voice defined a voice’s “distance-to-mean” (Figures 2A and 2C).

We first asked whether distance-to-mean was related to a behavioral measure of how distinctive each voice sounds to listeners. Normal adult listeners rated each voice for its perceived distinctiveness on a visual analog scale. Distinctiveness ratings, consistent across listeners, were significantly correlated with distance-to-mean (p < 0.001; Spearman’s ρ [CI95%] = 0.73 [0.62 0.81]; R^2^ = 0.53; Figure 2D; Figure S2A): voices located further away from their prototypical voice (i.e., more acoustically dissimilar) were perceived as more distinctive than closer (more acoustically similar) voices. This result, in line with previous observations [11], confirms the perceptual meaningfulness of our definition of distance-to-mean; it also provides an objective acoustical characterization of voice distinctiveness based on simple acoustical measures.

**Figure 3:**
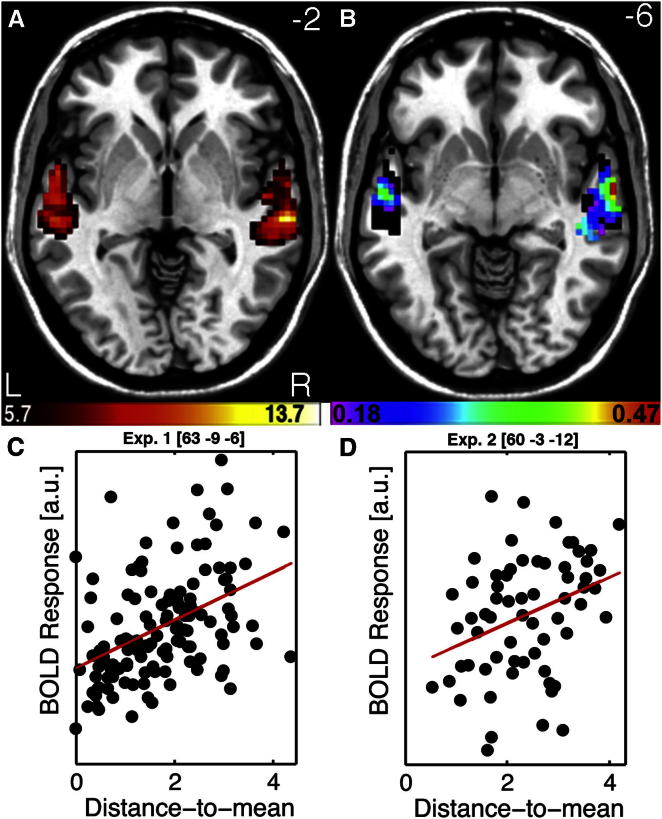
Cerebral Activity in Voice-Sensitive Cortex Correlates with Distance-to-Mean (A) TVA showing significantly greater fMRI signal in response to vocal versus nonvocal sounds at the group-level used as a mask for further analysis. Color scale indicates T values of the vocal versus nonvocal contrast. (B) Maps of Spearman correlation between beta estimates of BOLD signal in response to each voice stimulus and its distance-to-mean overlay on the TVA map (black). Color scale indicates significant ρ values (p < 0.05 corrected for multiple comparisons). Note a bilateral distribution with a maximum along the right anterior STS. See Figure S3 for correlation maps in 8 individuals. (C) Scatterplots and regression lines between estimates of BOLD signal and distance-to-mean at the peak voxel in Experiment 1. (E) Scatterplots and regression lines between estimates of BOLD signal and distance-to-mean at the peak voxel observed in Experiment 2. Scatterplots are shown for illustration only. See Audio S1 for example of stimuli used in Experiment 2.

We then asked whether distance-to-mean explains part of the cerebral response to a voice. We used fMRI to measure blood oxygenation level-dependent (BOLD) signal, an indirect index of neuronal activity [25], in the brain of healthy adult participants. Participants were first scanned in a 10 min “voice localizer” in order to localize the temporal voice areas (TVA). Group-level analysis highlighted a set of voxels in the classic location [1] along the middle portion of the superior temporal sulcus (mid-STS; Figure 3A; Table S2) with greater response to vocal than nonvocal sounds; this set of voxels (n = 1,096) defined the group-level TVA mask. BOLD signal was then measured while participants listened to the different voice stimuli (presented in runs of same-gender voices) and performed a pure tone detection task. We computed the Spearman correlation in each TVA voxel, across voice stimuli, between group-level beta estimates of BOLD signal and distance-to-mean; confidence intervals for the correlation values were estimated by using percentile bootstrap [26, 27]. Significance was assessed by using a permutation test at each voxel, and corrections for multiple comparison were based on the maximum significant threshold over the whole set of voxels (ρ = 0.18, Supplemental Experimental Procedures) [26, 28].

TVA voxels in both the left (LH) and right (RH) hemispheres showed significant positive correlations at the group level between BOLD signal estimates and distance-to-mean: voice stimuli at greater distance from the same-gender prototypical voice elicited greater BOLD signal (Figure 3B; Table S2). Significant rho values (p < 0.05; range = [0.18 0.47]) reached their maximum along right mid-STS (ρ [CI95%] = 0.47 [0.30 0.61]; MNI coordinates [63 −9 −6]; Figure 3C). Crucially, distances defined relative to a single, androgynous prototype instead of the two gender-specific prototypes (“distance-to-overall-barycentre”) never explained variance in BOLD signal better than distance-to-mean defined relative to gender-specific prototypes (Supplemental Information). Significant correlations between distance-to-mean and BOLD activity could also be observed at the single participant level, indicating the robustness of the effect (Figure S3). Outside the TVA, at a location close to primary auditory cortex (identified as the maximum of activity in the contrast of all sounds versus silence during the voice localizer: MNI coordinates [51 −6 0]), correlations between BOLD and distance-to-mean were just below significance (ρ [CI95%] = 0.17 [−0.01 0.35]). In addition, we found that distance-to-mean defined in simpler spaces with fewer dimensions also correlates with TVA activity, indicating that the effect is not restricted to that particular three-dimensional acoustical space (Figure S2B).

These results are consistent with the notion of norm-based coding of vocal identity, but they could also arise from neuronal adaptation effects, i.e., reductions in neuronal activity in response to repeated stimulation [14], that can in some conditions be mistakenly interpreted as prototype effects [13]. Further analyses were run on each voxel of the TVA to disentangle norm-based coding from adaptation effects. Briefly, different regressors were used to model adaptation at different time scales: (1) “distance-to-preceding-stimulus” to model short-term adaptation effects (∼4 s) occurring between consecutive stimuli during scanning, known to depend on physical similarity; (2) “distance-to-barycentre,” i.e., distance between each voice and the center of the (same-gender) voice cloud, that represent the average position of stimuli presented during an entire block (∼5 min) to model medium-term adaptation effects; and (3) “distance-to-overall-barycentre” (Figure 3D), i.e., to the average position of all male and female stimuli presented during the experiment (∼20 min) to model long-term adaptation. As expected, adaptation effects at different time ranges were found to occur in the TVA (Supplemental Information). Crucially, distance-to-mean resulted in significantly stronger correlation with BOLD signal than the adaptation regressors. Conversely, not a single voxel showed significantly larger correlation with either of the adaptation regressors (Supplemental Information). Thus, Experiment 1 provides strong evidence for a relation between TVA activity and acoustical similarity to (gender-specific) voice prototypes that is not simply explained by adaptation effects.

### Experiment 2

We next sought to replicate these results with different participants and stimuli. In Experiment 2, stimuli consisted of recordings of the word “hello” spoken by 32 male and 32 female adult speakers; i.e., recordings included only natural, unmanipulated stimuli. A new group of healthy adult volunteers was scanned while listening to individual stimuli and performing a pure tone detection task. Male and female prototypical stimuli were generated, following the same procedure as in Experiment 1 (Audio S1), to compute distance-to-mean values for each voice in the three-dimensional acoustical voice space (Table S1). Preprocessing steps and statistical analyses were similar to those of Experiment 1. Again, significant correlations between distance-to-mean and beta estimates of activity were found in several TVA voxels ranging from 0.25 to 0.36 with a maximum in the right hemisphere (ρ [CI95%] = 0.36 [0.13 0.55]; Figure 3D; Table S2). Thus, results of Experiment 1 appear generalizable to other participants and stimulus sets.

### Experiment 3

**Figure 4:**
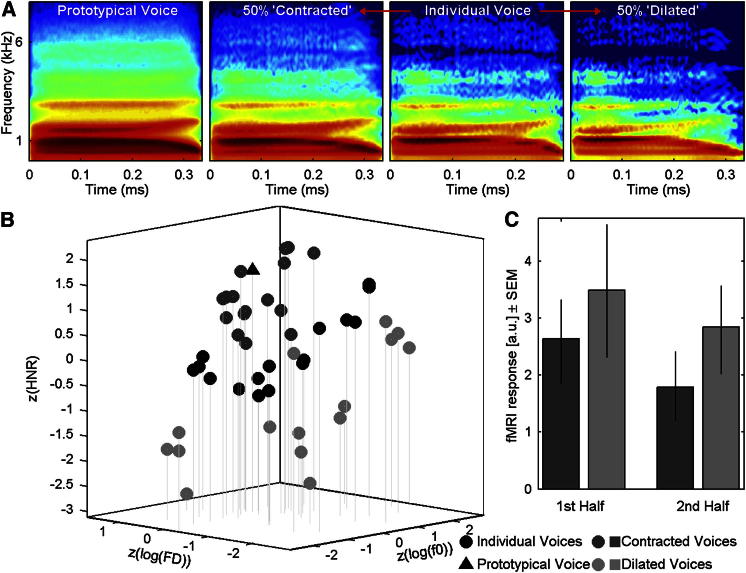
Manipulating Distance-to-Mean in Experiment 3 (A) An example voice stimulus (“had” spoken by an adult female speaker) is morphed with the female prototypical voice (left) to generate a “contracted” (moved 50% toward the prototypical voice in voice space) and a “dilated” (moved 50% away from the prototypical voice) versions of the voice: the contracted and dilated stimuli differ from the original voice by equal amounts of acoustical change, but dilated stimuli have greater distance-to-mean. See Figure S4 for an illustration of the morphing techniques used in Experiments 1 and 2. See Audio S2 for examples of stimuli used in Experiment 3. (B) Stimuli of Experiment 3 in acoustical voice space. Black disks represent original stimuli, dark gray discs represent contracted stimuli, light gray discs represent dilated stimuli, and the black triangle represents the prototypical voice. (C) fMRI response to contracted and dilated blocks for the first and second half of each block, at the peak voxel in RH. As predicted by the norm-based model, BOLD signal is greater in response to dilated than to contracted voices. Adaptation effects building up over the duration of a block (first half versus second half) do not interact with distance-to-mean. Error bars represent the 95% confidence interval.

Although the correlations observed in Experiments 1 and 2 support the norm-based model, they might still conceivably be caused by other, unknown factors in the stimulation related to both neuronal activity and distance-to-mean. More compelling evidence would be obtained by explicitly manipulating distance-to-mean and testing whether the differences predicted by the prototype-based model are observed. We ran a third experiment in which we used morphing to directly manipulate distance-to-mean while minimizing short-term adaptation effects by equating average distance-to-preceding-stimulus across conditions (Supplemental Experimental Procedures). For male and female voices independently, the 16 stimuli from the Experiment 1 stimulus set with intermediate distance-to-mean values were each morphed with the same-gender prototypical voice to generate a “contracted” (50% closer) and “dilated” (50% away) version of each stimulus (Figures 4A and 4B; Table S1; Audio S2). A new group of normal adult volunteers was scanned while listening to blocks of contracted or dilated stimuli and performing a pure tone detection task. At each TVA voxel, we computed the difference between the BOLD signals for “dilated” and “contracted” blocks. Statistical analyses, similar to those of Experiments 1 and 2, revealed differences in BOLD signal in TVA voxels (Table S2) consistent with the predictions of the norm-based model: voice stimuli induced greater TVA activity when morphed away from the prototype than when morphed toward the prototype by a same amount of acoustical change (significant threshold after correction for multiple comparisons = 0.58; range of significant differences = [0.58 1.00]). Here as well, adaptation effects did not simply explain the observed pattern of results (Figure 4C; Supplemental Information).

## Discussion

Like faces, voices can be used to identify a person, yet the neural bases of this ability remain poorly understood. Here we provide the first evidence of a norm-based coding mechanism in the auditory modality. Across three experiments using different participants and stimulus sets, we find that voices more distant from (dissimilar to) the same-gender prototypical voice in a three-dimensional f0-FD-HNR acoustical space are perceived as more distinctive, and elicit greater activity in the TVA, than closer (more similar) voices. The male and female prototypes appear to consist of ideally smooth versions of the population mean. We also demonstrate that this effect does not merely reflect adaptation effects occurring at different timescales.

These results are consistent with prior, mostly behavioral, evidence. A prototype model has been proposed to account for the variation in perceived distinctiveness between voices [7] and for the observation that speaker identification performance depends on the deviations of the speaker’s acoustic features from an estimated average [11]. The prototype model has recently received further support from behavioral studies using voice morphing [8, 10, 29]. In particular, voice identity aftereffects following adaptation with “antivoices” highlight a special status of the average voice [10]. By using fMRI and voice stimuli morphed between two familiar (learned) voice identities, Andics et al. also obtained evidence consistent with prototype-based coding of voices [4]. The present results are the first to demonstrate prototype-based effects both behaviorally and neurally in the context of a large number of unfamiliar voices.

Our findings confirm a functional organization of auditory cortex in which higher stages of cortical processing integrate acoustical features extracted by lower levels of cortex into more complex representations [30]. These representations appear to be referenced to an internal prototype. They also demonstrate that information on speaker identity is not only distributed over large zones of cortex as suggested by multivariate analyses [5] but can also be encoded locally, because correlations with distance-to-mean were observed at the level of individual TVA voxels. Whether similar mechanisms can be observed for other categories of auditory objects remains to be determined.

The results bring to light interesting similarities in coding mechanisms across sensory modalities [31]. Converging evidence from psychophysical, electrophysiological, and neuroimaging studies indeed indicates that individual faces are represented in part as a function of their distance to a prototypical, average face in a multidimensional “face space” [32, 33, 34, 35]. Although the exact nature of the face prototype remains unclear [36], this sparse coding mechanism is thought to offer several advantages including a minimization of energy consumption in response to natural stimulation and an elegant solution to overcome the problem of certain transformations associated with, e.g., viewpoint change or aging. Despite the highly different nature of the sensory input from faces and voices, an analogous mechanism appears to be used to represent a person’s identity across sensory modalities. This does not imply that all cortical processing is similar across vision and audition but illustrates a parsimonious principle of brain organization given the similar nature of the computational problems posed by face and voice identity processing and the fact that information has to be integrated across senses in everyday life [37].

Important information was obtained on the characteristics of the prototypical stimuli, a question that still eludes research on face perception [36]. There are two voice prototypes: one male and one female. A model defining distance-to-mean relative to a single, androgynous prototype explained virtually no variance. The male and female prototypical voices appear well approximated by the morphing-generated average of many same-gender voices, a process resulting in voices with (gender) average f0 and formant frequencies but with high HNR values; i.e., a very common voice but ideally regular and flawless, perceived as highly attractive by listeners [8, 9]. This unanticipated feature of the prototypical voices allows demonstrating norm-based coding and differentiates its effects from those of adaptation occurring over the medium- to long-term (cf. Supplemental Information).

We find that distance-to-mean defined in simpler (two- or one-dimensional) spaces also predicts TVA activity and perceptual ratings of distinctiveness, indicating that the f0-FD-HNR acoustical space used here is not the only valid voice space. The “true” voice space is likely to include a larger number of more complex dimensions, consistent with the intricacy of the voice production apparatus and the many associated acoustical dimensions [15, 17, 18, 19] (Figure 1; Figures S2A and S2B). Nevertheless, the f0-FD-HNR space appears an adequate approximation of the true voice space allowing the estimation of a voice’s distance-to-mean from a small number of easily measured acoustical variables.

Note that our results were obtained in the context of a large number of unfamiliar voices. Whether similar mechanisms are involved in coding the identity of familiar voices remains to be established [38], although clinical studies of voice perception [39, 40, 41] and face perception studies [42] suggest qualitatively different mechanisms for familiar stimuli. Likewise, the extent to which these results generalize to longer, more complex utterances representative of more natural conditions of conversation remains to be investigated.

These results in turn lead to a range of important new questions. Are the prototypes innate, stored templates? Or are they susceptible to environmental and cultural influences? If yes, to what extent? Could the prototype consist of an average of all voices experienced during one’s life, in which case the frontier between prototype-based coding and long-term adaptation would become blurry? Can similar coding principles be observed in the brain of nonhuman primates, as has been found for faces [35]? While the answers to these questions are yet unknown, our results provide the first evidence of norm-based coding of voice identity in human auditory cortex, a finding with potentially useful applications in voice and speech signal processing [43]. These studies bring to light similarities in encoding strategies between sensory modalities, but also define important characteristics of the internal voice prototypes: they are ideally smooth versions of the male and female population means.

## Supplemental Information

Document S1. Figures S1–S4, Table S2, Supplemental Data, and Supplemental Experimental Procedures

Table S1. Acoustic Measures of the Original and Prototypical Voices used in Experiments 1 and 2, as well as All Contracted and Dilated Voices used in Experiment 3 Dur, duration (ms); f0, fundamental frequency (Hz); Std(f0), SD of f0 (Hz); Int, intonation measured as the difference in f0 between the first and last voiced frames (Hz); F1–F4, frequencies of the first four formants (Hz); FD, formant dispersion (Hz); H1–H2, power difference between the first two harmonics (dB); HNR, harmonic-to-noise (dB); Jitter, 100 times jitter value (5); alpha, alpha ratio; Loud, loudness (dB).

Audio S1. Voices of Experiment 2 One audio file displaying four different voices, related to Figure 2. Voices 1 and 2 are examples of original male and female voice stimuli respectively; voices 3 and 4 are the male and female prototypical voice stimuli, respectively.

Audio S2. Voices of Experiment 3 The clips correspond to the stimuli shown in the spectrograms of Figure 4, related to Figure 4. One audio file displaying four different voices: (1) prototypical female voice, (2) original voice stimulus, and (3) original voice “contracted” and (4) “dilated” versions.
