# Supplementary material for: Norm-Based Coding of Voice Identity in Human Auditory Cortex
Source: Curr Biol. 2013 Jun 17;23(12):1075–80. doi: 10.1016/j.cub.2013.04.055 (PMC3690478; doi:10.1016/j.cub.2013.04.055)
Supplement: Document S1. Figures S1–S4, Table S2, Supplemental Data, and Supplemental Experimental Procedures [file mmc1.pdf]

Current Biology, Volume *23*

Supplemental Information

Norm-Based Coding of Voice Identity

in Human Auditory Cortex

Marianne Latinus, Phil McAleer, Patricia E.G. Bestelmeyer, and Pascal Belin

Supplemental Inventory

1. Supplemental Figures and Tables

Figure S1, Related to Figure 1

Figure S2, Related to Figure 2

Figure S3, Related to Figure 3

Figure S4, Related to Figure 4

Table S2

2. Supplemental Data

3. Supplemental Experimental Procedures

4. Supplemental References

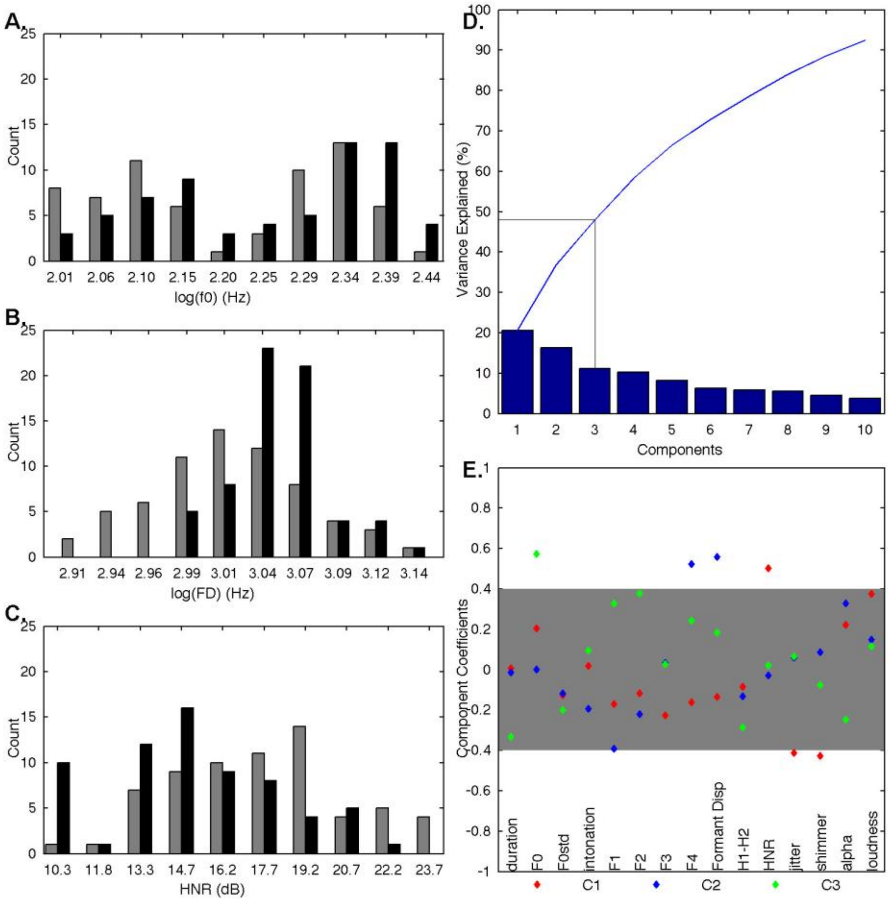

Figure S1. Acoustical Analyses, Related to Figure 1

(A,B,C) Distributions of acoustical values in Experiments 1 and 2. Individual acoustical measures, z-scored by gender, are shown for log(f0), log(FD) and HNR for stimuli of Experiment 1 (gray bars) and Experiment 2 (black bar).

(D) Principal component analysis (PCA) of acoustical measures for stimuli of Experiment 1. The first three components explained nearly half (48.1%) of the variance in acoustical measures with three easily interpretable dimensions.

(E) The scatterplot represents the loading on each acoustical parameter for the first three components of the PCA. To highlight parameters with higher weights, a shaded area was added for weights between -0.4 and 0.4. The first component (explaining 20.7% of the variance) reflected spectro-temporal regularity as measured, in particular, by HNR; the second component largely reflected FD (16.1% of the variance) while the third component was most strongly related to average f0 (11.3% of the variance).

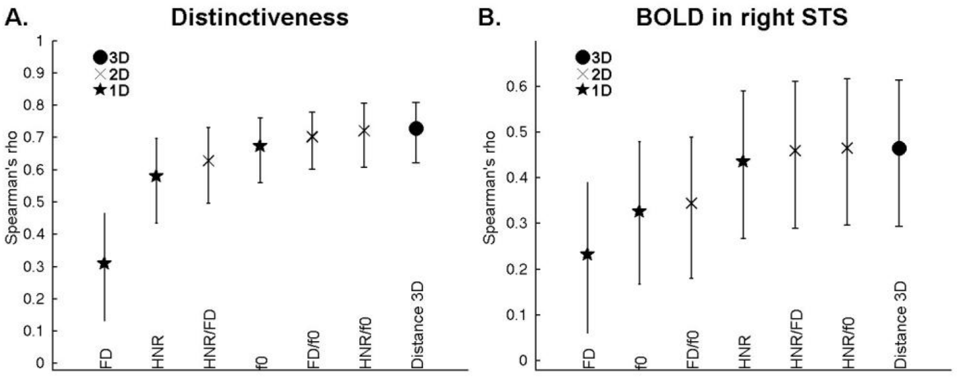

Figure S2. Correlations with Distance-To-Mean Measured in Spaces with Fewer Dimensions, Related to Figure 2

(A) Correlations between distinctiveness ratings and distance-to-mean defined in uni- (star) or bi- (cross) dimensional spaces, as well as the 3D f0-FD-HNR space (disc). Bars represent 95% confidence intervals estimated using percentile bootstrap.

(B) Spearman's ρ of the correlation between beta estimates of BOLD activity at the voxel with the highest correlation in the right hemisphere and distance-to-mean defined in uni- (star) or bi- (cross) dimensional spaces, as well as the 3D f0-FD-HNR space (disc). Bars represent 95% confidence intervals estimated using percentile bootstrap. Correlations between TVA activity and distance-to-mean are significant for bi- as well as uni-dimensional spaces, indicating that the f0-FD-HNR acoustical space used here is not the only valid voice space. Note results are provided at the voxel with the highest correlation in RH for illustration, other voxels of the TVA could have different patterns of response.

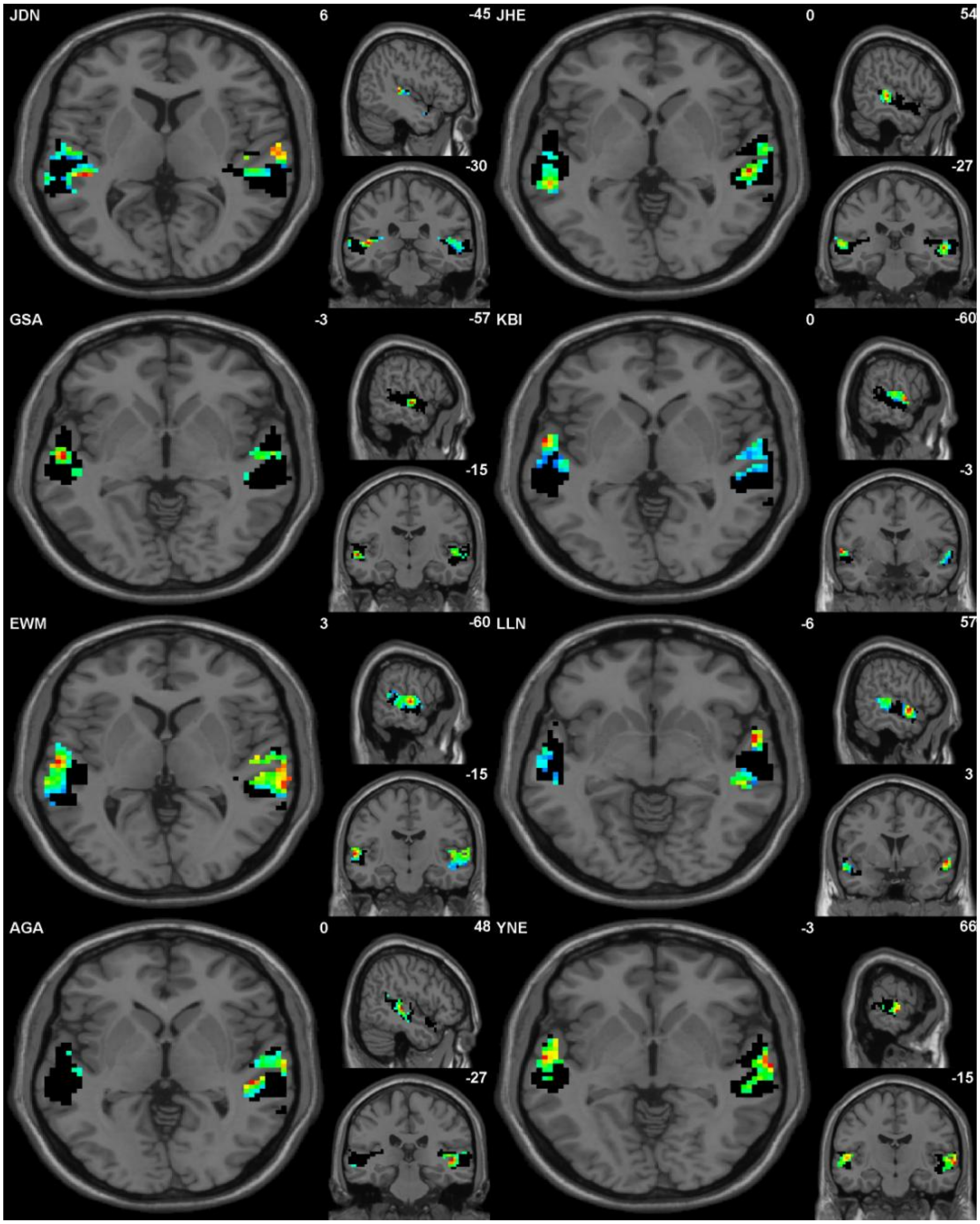

Figure S3. Correlations between TVA BOLD Estimates and Distance-to-Mean at the Single Participant Level in Experiment 1, Related to Figure 3

Maps of Spearman correlation between beta estimates of BOLD signal and distance-to-mean are shown in 8 participants overlaid in color scale (threshold: p=0.05) over the TVA (black) and anatomical sections of a template. MNI coordinates of the slices shown are indicated in numbers. Letters refer to participant codes.

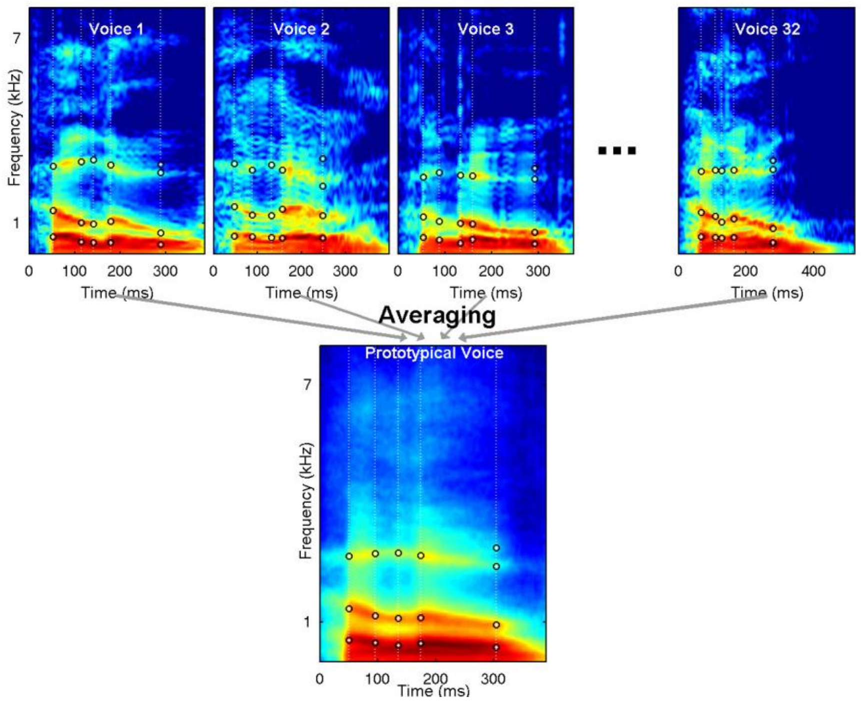

Figure S4. Voice Averaging in Experiment 2, Related to Figure 4

Top: Spectrograms of example voice stimuli: female speakers uttering the word “hello”. Time-frequency landmarks correspond to frequencies of the first three formants (plus the fourth for landmarks at the end of phonation) measured at: onset of phonation; offset of first phoneme; onset of second phoneme; offset of formant transition in second phoneme; offset of phonation.

Bottom: spectrogram of the prototypical female stimulus generated by averaging 32 stimuli, showing average positions of the time-frequency landmarks.

Table S2. Coordinates of fMRI Peaks for Voice Localiser, and LH and RH Maximums for Experiments 1 to 3

Hemisphere		x	y	z	T/rho/Diff	No voxels
Voice Localizer						
Left	STC	-60	-27	3	13.8	537
		-60	-12	6	12.6	
		-60	-6	0	12.2	
Right	STC	63	-24	-3	13.1	559
	MTC	57	-33	0	12.1	
	sTP	48	9	-18	10.5	
Experiment 1						
Left	mid-STC	-60	-12	-3	0.4	
Right	mid-STC	63	-9	-6	0.47	
	sTP	51	12	-15	0.23	
Experiment 2						
Left	pSTC	-57	-45	12	0.27	
Right	pSTC	48	-48	15	0.36	
	aSTC	60	-3	-12	0.39	
Experiment 3						
Left	mid-STC	-57	-15	9	1.003	
Right	mid-STC	63	-9	6	0.963	

STC: Superior Temporal Cortex; MTC: middle Temporal Cortex; sTP: superior Temporal Pole; pSTC: posterior STC; aSTC: anterior STC.

Supplemental Data

To further explore adaptation effects occurring at different time range, we computed at each TVA voxel the Spearman correlations between BOLD estimates of TVA activity and three different regressors modeling adaptation at three different time scales: (i) distance-to-overall-barycenter (long-term), (ii) distance-to-barycenter (medium-term), (iii) distance-to-preceding-stimulus (short-term). We then computed differences between Spearman's ρ observed for each regressors and that of distance-to-mean; multiple comparisons correction was again applied using the maximum significant threshold over the whole set of voxels.

Long-term adaptation effects could gradually build up over the history of the scanning session whereby a stimulus more similar to the ensemble of all stimuli already presented elicits smaller response [4]. This effect can be modeled by the "distance-to-overall-barycenter" in voice space, i.e., distance to the average location of all stimuli (male as well as female) presented during scanning: a point of voice space with an intermediate position between male and female voices. After multiple comparisons correction (mcc), significant correlations between BOLD activity and distance-to-overall-barycenter were found in the TVA (ρ range: [0.19 0.24]). Crucially, no voxel showed a significantly greater Spearman's ρ with distance-to-overall-barycenter than with distance-to-mean; on the contrary, distance-to-mean correlated significantly stronger with TVA activity than distance-to-overall-barycenter (475 voxels; mcc threshold = 0.31; ρ difference range [0.31 0.61]).

Adaptation effects potentially occurring at the intermediate time scale of single runs, during which voices of a particular gender were heard, were examined by defining distances from the barycenter of same-gender stimulus clouds (i.e., of the stimuli presented within a single run – referred to as distance-to-barycenter). Correlations between BOLD estimates and distance-to-barycenter of same-gender stimuli were significant on several TVA voxels (ρ range: [0.18 0.33]) suggesting the presence of adaptation effects at the level of single runs. However, variance in TVA activity was again better explained by distance-to-mean (64 voxels; mcc threshold = 0.20; ρ difference range [0.21 0.31]).

Finally, adaptation effects can also occur at a shorter time scale: considerable evidence indicates that neuronal activity in sensory cortex is strongly affected by the physical similarity between the current stimulus and the stimulus presented immediately before [2, 3, 44, 45]. This short-term adaptation effect can be modeled by the "distance-to-preceding-stimulus": a stimulus closer in voice space to the stimulus presented immediately before during scanning will be subject to greater short-term adaptation. Distance-to-mean and distance-to-preceding-stimulus were strongly correlated in our paradigm (ρ [CI95%] = 0.67 [0.54 0.76]; $p<0.001$): during scanning, voice stimuli with small distance-to-mean tended on average to be preceded by stimuli that were closer in voice space than voices with a larger distance-to-mean. Again, correlations between BOLD estimates and distance-to-preceding-stimulus were found on several TVA voxels (ρ range: [0.18 0.39]) suggesting the presence of short-term adaptation effects. While 49 voxels showed significantly greater correlations with distance-to-mean than with distance-to-preceding-stimulus (mcc threshold = 0.17; ρ difference range [0.17 0.25]), no single voxel showed the reverse pattern of a greater correlation with distance-to-preceding-stimulus than distance-to-mean.

This finding is confirmed by a supplementary analysis restricted to voices with small distances-to-mean (0-2; 58% of the stimuli). For those voices, the correlation between distance-to-mean and distance-to-preceding-stimulus was not significant (ρ [CI95%] = -0.14 [-0.38 0.09];

p=0.23), i.e., these stimuli were as likely to be preceded by acoustically similar than by dissimilar stimuli, and were hence subject to similar short-term adaptation effects. Yet, correlations between distance-to-mean and TVA activity remained significant for those voices in 225 TVA voxels (ρ range: [0.24 0.47]). Taken together, results from Experiment 1 clearly demonstrate that distance-to-mean explains activity variation in voice-sensitive cortex over that imputable to adaptation occurring at different time scales.

In Experiment 3, the influence of longer-term adaptation effects potentially building over the block duration was examined by separately analyzing the response to stimuli presented during the first vs. second half of each block, at the peak voxel in each hemisphere ([46], Fig. 4C). In both hemispheres, "dilated" voices induced greater activity than "contracted" voices for both the first (RH: difference [CI95%]=0.86 [0.28 1.42]; LH: difference [CI95%]=0.85 [0.61 1.08]) and second half (RH: difference [CI95%]=1.07 [0.69 1.43]; LH: difference [CI95%]=1.154 [0.594 1.638]) of the blocks. To assess whether time had a significant effect, we computed the difference between "dilated" and "contracted" blocks independently for the first and second halves of the blocks; we then calculated the difference of the differences ([Dilated1-Contracted1]– [Dilated2-Contracted2]) at the peak voxel in each hemisphere (LH, RH: -0.30; -0.22). It was not significantly different from 0 (threshold = LH: [-0.58 0.58]; RH: [-0.55 0.55]), ruling out an explanation of the observed differences in terms of longer-term adaptation effects building up over the course of each block.

Supplemental Experimental Procedures

Participants

Participants were healthy adult volunteers recruited from the student population of the University of Glasgow (Experiment 1 – ratings: N=15, 4 males, 28.1±4.6 years old; Experiment 1 – fMRI: N=20, 9 males, 23.2±3.7 years old; Experiment 2: N=21, 10 males, 24.3±5.4 years old; Experiment 3: N=7, 4 males, 25.9±3.5 years old). They all reported normal audition, gave written informed consent and were compensated at the rate of £6/h. The study was approved by the ethics committee of the University of Glasgow.

Stimuli

In Experiment 1, voice stimuli (n=126), previously used in studies of vocal attractiveness [8, 9], consisted of recordings of female (n=32) and male (n=32) speakers uttering the syllable "had" [24], as well as voice composites (n=62, 31 per gender) generated by averaging 2, 4, 8, 16 or 32 voices via morphing (cf. Morphing). Stimuli were edited using Adobe Audition (Adobe Systems Inc.) to remove the release burst in the final 'd' and normalized for energy (RMS) using in-house code in Matlab (The MathWorks, Inc.). In Experiment 2, stimuli (n=64) consisted of recordings of female (n=32) and male (n=32) Scottish speakers uttering the word "hello". Speakers were digitally recorded (16bit, mono, sampling rate: 44.1kHz) while reading an unfamiliar text in a sound-attenuated booth. They were instructed to read the passage in a neutral tone. The word "hello" was extracted from each recording, and normalized for overall energy (RMS; **Audio clip S1**). In Experiment 3, original stimuli consisted of the 32 (16 male, 16 female) natural stimuli from Experiment 1 with intermediate distance-to-mean values. They were morphed with the prototypical voice in order to move them 50% closer ("contracted") or 50% away ("dilated") from the same-gender prototype and modify their duration and temporal landmarks to those of the same-gender average (**Fig. 4; Audio clip S2**).

Morphing

Voice morphing and averaging was performed using STRAIGHT [12] in Matlab. STRAIGHT performs an instantaneous pitch-adaptive spectral smoothing in each stimulus for separation of contributions to the voice signal arising from the glottal source vs. supra-laryngeal filtering. A voice stimulus is decomposed by STRAIGHT into five parameters: f0, frequency, time, spectro-temporal density and aperiodicity that can be manipulated and combined across stimuli independently of one another. Time-frequency landmarks to be put in correspondence across voices during morphing were manually identified in each stimulus. For stimuli used in Experiments 1 and 3, landmarks corresponded to frequencies of the first three formants at onset of phonation, at onset of formant transition, and at offset of phonation (**Fig. 2B**). For stimuli used in Experiment 2, landmarks corresponded to frequencies of the first three formants (plus the fourth for landmarks at the end of phonation) measured at: onset of phonation; offset of first phoneme; onset of second phoneme; offset of formant transition in second phoneme; offset of phonation (**Fig. S4**).

Prototypical voices of each gender were generated by morphing all 32 same-gender stimuli based on the interpolation of the individual time-frequency landmark templates each weighted with a weight of 1/32. In Experiment 3, “contracted” and “dilated” stimuli were generated by morphing each stimulus with the same-gender prototypical voice with weights of 0.5 and 0.5 each for the “contracted” stimuli, and of -0.5 (prototypical voice) and 1.5 (individual voice) for the “dilated” stimuli (**Fig. 4**). This interpolation was done for all parameters except for time, for which each voice was given a weight of 1/32 resulting in average duration and temporal landmarks for all stimuli.

Acoustical Measures

Acoustical measures of all stimuli (**Table S1**) were performed using Praat [47]. Measured parameters consisted of: the mean fundamental frequency (f0 (Hz)), the standard deviation of f0 (Hz), the mean frequency of the first four formants (F1-F4 (Hz)), formant dispersion, i.e. the average frequency difference between formants (FD (Hz)), the energy difference between the first two harmonics (H1-H2 (dB)), the alpha ratio (i.e., the ratio of mean energy within low (0-1kHz) vs. high frequencies (1-5 kHz) computed from the long-term average spectrum), the mean harmonics-to-noise ratio (HNR (dB)), shimmer (local – dB) and jitter (relative average perturbation – %), a measure of intonation obtained by computing the difference between the f0 of the last vs. the first voiced frame (Hz), as well as amplitude (loudness – dB) and sound duration (ms). All frequency-related parameters were expressed on a log-scale.

Ratings

Perceived distinctiveness of stimuli in Experiment 1 was assessed with an analogue visual scale ranging from “Not at all distinctive” to “Extremely distinctive”. Original and composite voices (n=63 per gender) were presented twice in a pseudo-random order. Female and male voices were rated in two separate blocks presented in a counterbalanced order. Ratings were individually z-scored using the mean and standard deviation of ratings for same-gender natural voices. Inter-rater agreement was high (Cronbach’s alpha: .94 for female voices and .90 for male voices).

fMRI Scanning

Images of blood oxygenation-level dependent (BOLD) signal were acquired on a 3T Tim Trio scanner (Siemens, Erlangen). Whole-brain T1-weighted anatomical scans were performed using a fast gradient echo sequence (MPRAGE; 192 axial slices; voxel size: 1x1x1 mm; 256x256 matrix). T2*-weighted functional scans were acquired using an echo-planar imaging (EPI) sequence (32 axial slices; voxel 3x3x3 mm; 70x70 matrix; 0.3 mm gap between slices) and an interleaved ascending order. Auditory stimulation was delivered binaurally via an MRI-compatible headphone system (NordicNeuroLab Inc.) at a level of 80 dB (SPL) and subject responses were recorded via optic hand-held response buttons (Current Designs Inc.).

During the voice localizer scan (TR = 2s; TE = 30ms; 310 volumes, 10 minutes), participants were instructed to listen passively to 10s blocks of either vocal sounds (n=21) or non-vocal sounds (n=21) interspersed with silent blocks (n=21) presented in a pseudo-randomized order over the continuous scanner background noise. Each block started with 2s of silence followed by 8s of different stimuli from the same category. Stimuli are described in detail elsewhere [1]. Briefly, vocal sounds consist of brief excerpts of speech (syllables, words and sentences in different languages, etc.) and non-speech (laughs, sighs, coughs, etc.) vocalizations. Non-vocal sounds consist of industrial and environmental sounds, as well as animal vocalizations. Stimuli are available at: http://vnl.psy.gla.ac.uk/resources.php.

Experiment 1 consisted of four runs (2 runs per gender) of 86 trials each: 63 voices of the same gender uttering the syllable “had” (32 natural and 31 composites voices), 7 pure tones (1000Hz) and 9 additional silences. Stimuli within each run were presented in a pseudo-random order at a 4s stimulus-onset-asynchrony (SOA) during the 500ms silent gap between consecutive EPI volume acquisitions (TR = 2s, TA = 1.5s, TE = 30ms; 180 volumes, 6 min per run). Participants were instructed to listen to the stimuli and press a button every time they heard a pure tone. The pure tone detection task was designed to keep the subject’s attention alert while not focused on any particular aspect of the voice stimulation.

Experiment 2 consisted of a single run during which 12 blocks of male voices and 12 blocks of female voices were presented each separated by 18 seconds of no stimulation while the participant was engaged in a pure tone detection task. In each block, each of the 32 stimuli of one gender was presented once, as well as an average of 2 pure tones per block, at a 3s SOA during the 1s silent gap between consecutive EPI acquisitions (TR=3s, TA=2s, TE=30ms; 990 volumes, 48 minutes). Block presentation order and stimulus presentation order within blocks were randomized for each participant.

Experiment 3 consisted of a single run during which 32 blocks of stimuli were presented each separated by 30s periods of no stimulation while the participant was engaged in a passive listening task. Blocks belonged to one of four conditions (8 blocks per condition) defined by a 2x2 factorial design with a factor gender (male/female) and a factor distance (contracted/dilated). During each block, the 16 stimuli for that condition were presented at a 2s SOA during the 500ms gap between consecutive EPI acquisitions (TR=2s, TA=1.5s, TE=30ms; 1020 volumes, 34 minutes). Order of block presentation was randomized between participants; however stimuli order within each block was arranged such that distance-to-preceding-stimulus values did not differ significantly between Contracted and Dilated conditions. This was achieved by selecting stimulus presentation orders that maximized distance-to-preceding-stimulus for the Contracted blocks (by selecting as the next voice the one most distant from the current voice) and that

minimized it for the Dilated blocks (by selecting the next closest voice): distance-to-preceding stimulus (mean ± s.d.): contracted = 1.53± 0.036; dilated = 1.55±0.42 ($t(30)=-1.5$, $p=0.14$)

fMRI Analysis

EPI images were pre-processed according to standard procedure using SPM8 (Wellcome Department of Imaging Neuroscience; http://www.fil.ion.ucl.ac.uk/spm): functional scans were motion-corrected, co-registered with the within-session anatomical scan, normalized to the Montreal Neurological Institute (MNI) template, and smoothed using a Gaussian function with the full-width at half-maximum of 8mm isotropic. Functional data were analyzed in a two-level random-effects design.

In the voice localizer, first-level analysis involved a design matrix containing separate regressors for vocal and non-vocal sounds, plus realignment parameters to account for residual motion artifacts. These regressors contained boxcar functions representing the onset and offset of stimulation blocks convolved with a canonical hemodynamic response function (HRF). Using the modified general linear model, parameter estimates for vocal and non-vocal sounds were estimated and used to create a contrast image of the difference between vocal and non-vocal sounds. Contrast images from each participant were entered into a second-level Student's T-test analysis. The TVA mask was created using a voxel threshold of $p<10^{-5}$, and a cluster threshold of 125 voxels (FWE corrected); it included 1096 voxels.

Data from Experiment 1 was previously analyzed in a study of the cerebral correlates of perceived vocal attractiveness at the whole-brain level [9]. Here, analyses focused on TVA voxels and used distance-to mean defined in f0-FD-HNR space. First-level analysis involved a design matrix containing a separate regressor for each voice stimulus (n=126: 64 natural and 63 composite voices), plus covariates modeling realignment parameters. These regressors contained the onset time of the voice stimulus convolved with a canonical HRF. Parameter estimates for each voice stimulus were estimated and used to create individual contrast images for each voice stimulus (126 contrasts) across the brain volumes. Voxel-based random effects analysis was performed on the individual contrasts using Student's T-tests. Beta estimates for each voice stimulus (at the first and second level), extracted from each voxel of the TVA mask, were used in a series of Spearman correlations.

In Experiment 2, individual contrast images for each voice stimulus (n=64) across the brain volume were generated for each participant following a procedure similar to that of Experiment 1. These individual contrast images were co-registered to the voice localizer scan. Voxel-based random effect analysis was then performed on the co-registered individual contrasts using Student's T-tests. Beta estimates were then extracted from each voxel of the TVA mask, and entered in a series of Spearman correlations; subsequent analysis steps were as in Experiment 1.

In Experiment 3, the first-level analysis involved a design matrix containing a separate regressor for each condition (model 1). Effects of time on block were modeled in another model (model 2) with condition entered in half-blocks. These regressors contained boxcar functions representing the onset and offset of stimulation blocks convolved with a canonical HRF. Parameter estimates for each condition were estimated and used to create contrast images of each condition relative to baseline. These individual contrast images were co-registered to the voice localizer scan. Beta estimates were extracted from the voxels included in the TVA mask, and

differences between "dilated" and "contracted" blocks were calculated for the whole block (model 1) and for the first and second half of each block (model 2).

Statistical Analysis

We have opted to use a robust statistics framework for the analysis of the relation between stimulus characteristics and fMRI signal [25, 26] as this is more robust to outliers, produces more accurate estimates of significance level based on confidence intervals drawn from distribution of the data themselves and not requiring the assumptions of Random Field Theory. In Experiment 1 and 2, confidence intervals (CI) around Spearman's ρ values were estimated at the peak voxels by computing ρ after bootstrapping with replacement (10000 bootstraps) of the beta/distance-to-mean pairs [27]. P values were estimated using permutation tests providing a robust estimation of the significance of the reported correlations. Statistical assessment of the differences between correlations was performed by calculating the observed differences in ρ then calculating theoretical differences using permutation tests. In Experiment 3, CI of the differences was calculated at all voxels of the TVA mask using a percentile bootstrap (10000 bootstraps); permutation tests allowed the estimation of the significance threshold. To assess whether time had a significant effect, we computed the difference between "dilated" and "contracted" blocks independently for the first and second halves of the blocks; we then calculated the difference of the differences ([Dilated1-Contracted1]– [Dilated2-Contracted2]) at the peak voxel in each hemisphere. Percentile bootstrap was used to determine the CI of the difference of differences, while permutation tests allowed the estimation of the significance threshold. In all statistical tests, corrections for multiple comparisons performed by comparing the observed values to the maximum significant threshold over the whole set of voxel [26, 28].

Supplemental References

44. Aguirre, G.K. (2007). Continuous carry-over designs for fMRI. Neuroimage *35*, 1480-1494.
45. Panis, S., Wagemans, J., and Op de Beeck, H.P. (2011). Dynamic norm-based encoding for unfamiliar shapes in human visual cortex. J Cogn Neurosci *23*, 1829-1843.
46. Davidenko, N., Remus, D.A., and Grill-Spector, K. (2011). Face-likeness and image variability drive responses in human face-selective ventral regions. Human brain mapping.
47. Boersma, P., and Weenick, D. (2001). Praat, a system for doing phonetics by computer. Glot International *5*, 341-345.
